# Optimal experimental conditions for Welan gum production by support vector regression and adaptive genetic algorithm

**DOI:** 10.1371/journal.pone.0185942

**Published:** 2017-10-09

**Authors:** Zhongwei Li, Xiang Yuan, Xuerong Cui, Xin Liu, Leiquan Wang, Weishan Zhang, Qinghua Lu, Hu Zhu

**Affiliations:** 1 College of Computer and Communication Engineering, China University of Petroleum, Qingdao 266580, Shandong, China; 2 College of Chemistry and Materials, Fujian Normal University, Fuzhou 350007, China; Xiamen University, CHINA

## Abstract

Welan gum is a kind of novel microbial polysaccharide, which is widely produced during the process of microbial growth and metabolism in different external conditions. Welan gum can be used as the thickener, suspending agent, emulsifier, stabilizer, lubricant, film-forming agent and adhesive usage in agriculture. In recent years, finding optimal experimental conditions to maximize the production is paid growing attentions. In this work, a hybrid computational method is proposed to optimize experimental conditions for producing Welan gum with data collected from experiments records. Support Vector Regression (SVR) is used to model the relationship between Welan gum production and experimental conditions, and then adaptive Genetic Algorithm (AGA, for short) is applied to search optimized experimental conditions. As results, a mathematic model of predicting production of Welan gum from experimental conditions is obtained, which achieves accuracy rate 88.36%. As well, a class of optimized experimental conditions is predicted for producing Welan gum 31.65g/L. Comparing the best result in chemical experiment 30.63g/L, the predicted production improves it by 3.3%. The results provide potential optimal experimental conditions to improve the production of Welan gum.

## Introduction

Welan gum is a kind of polysaccharide, which is one of the secretions of Alcaligenes sp.NX-3 strain. It has good stability, ideal thickening property, unique shear thinning property, good suspension and emulsification, and assured safety, and can be used in oil drilling with its unique shear-thinning properties. Finding optimal experimental conditions to maximize the production of Welan gum is paid growing attentions. This can process the production of Welan gum industrially. In 2014, producing Welan gum fermentation in laboratory is achieved in [1], where cyperus beans are used as raw materials, protein and hydrolysis as substrate. After that, Bacillus foecalis alkaligenes are designed as starting bacterial strain, to optimize the yield process of Welan gum by response surface method [2].

It is found that many factors affecting the production of Welan gum, such as glucose, yeast, liquid volume, PH vale, temperature, which contribute the experimental conditions of producing Welan gum. To find the optimal experimental conditions, we need to consider the following aspects:

1. function of each factor;
2. interaction between each pair of factors;
3. relationship among all the factors.

In 2010, Li et al used the batch fermentation experiment data of Welan gum’s starting bacterial strain Alcaligenessp.CGMCC2428 to carry out the dynamic model research, implemented fermentation process of Welan gum optimization control [3]. In 2016, JMP statistical analysis software was used to optimize the fermentation medium of Welan gum by Alcaligenes sp.Y5. With the optimized experimental conditions, the production of Welan gum was increased from 15.72 g/L to 26.58 g/L, with an increment of 69.08% [4].

Recently, many significant artificial intelligent algorithms and data processing strategies has been applied on data mining, such as a self-adaptive artificial bee colony algorithm based on global best for global optimization [5], the public auditing protocol with novel dynamic structure for cloud data [6], privacy-preserving smart semantic search method for conceptual graphs over encrypted outsourced data [7], a privacy-preserving and copy-deterrence content for image data processing with retrieval scheme in cloud computing [8], strategy solving NP problems such as subset sum problem based on SN P systems [9], Apriori algorithm based on tissue-like P systems [10], split clustering algorithm based on P systems on simplices [11], spatial clustering algorithm based on DNA model [12], PSO algorithm based on dynamic niche technology [13] and machine learning method have been applied for experimental condition design, see. e.g. a secure and dynamic multi-keyword ranked search scheme over encrypted cloud data [14]. In this work, we presents a hybrid computational method to optimize experimental conditions for producing Welan gum with data collected from experiments records. Specifically, Support Vector Regression (SVR) is used to model relationship between Welan gum production and experimental conditions, and then adaptive Genetic Algorithm (AGA) is used to search optimized experimental conditions. As results, a mathematic model of predicting production of Welan gum from experimental conditions with accuracy rate 88.36% is obtained, a class of optimized experimental conditions is designed to produce Welan gum 31.65g/L. Comparing the best results in chemical lab 30.63g/L, the predicted production can be improved by 3.3%. The result provides a potential experimental conditions by data mining to improve the production of Welan gum in the lab.

## Related technologies

In this section, the two main methods used, Support Vector Regression (SVR) and adaptive Genetic Algorithm (AGA), are briefly recalled.

Here, we choose the SVR method mainly because of our limited samples. First of all, as for the regression of a small amount of samples, SVR has many advantages, such as a few adjusted parameters and fast arithmetic speed, etc. Secondly, the final decision function of SVR is determined by only a small number of support vectors. Finally, the computational complexity depends on the number of support vectors, not the dimension of the sample space, which also reflects that the robustness of the SVR method is better.

Genetic algorithm is a global search algorithm, which have a good reference for our problems. However, the traditional genetic algorithm still needs to be improved in terms of global search ability and convergence speed. The adaptive Genetic Algorithm we adopt can improve these two aspects to a certain extent. In the case of crossover probability, the AGA method can enable the crossover probability to vary with the evolution process and give the same crossover ability to the individuals of the same generation population, so as to realize the global search ability better. In the case of mutation probability, according to the fitness value of each individual to be mutated, the AGA method can make the mutation probability adaptively change with the evolutionary process.

### Support vector regression

Support Vector Machine (SVM) is known as a kind of machine learning method for classification proposed in 1995 [15], has been widely used in biological data processing [16–18] and bioinformatics [19–23]. It focuses on doing classification with seeking structured minimum risk to improve the generalization ability of learning machine and minimizing empirical risk and confidence limit [24, 25], thus achieving good statistical law under the condition of the less statistical sample size. In general, it is a kind of two-category model, the basic model is defined as the feature space interval on the maximum linear classifier. The learning strategy of SVM is to maximize the interval, which finally can be converted into a convex quadratic programming problem.

Support Vector Regression (SVR) is developed based on SVM for dealing with regression forecasting problems [26, 27]. Some basic concepts of SVR are briefly recalled.

Given a set of training data {(**x**_1_, *y*_1_), (**x**_2_, *y*_2_), …, (**x**_*l*_, *y*_*l*_)}, *R*^*n*^ × *R*, where **x**_*i*_ denotes the input samples, *y*_*i*_ is the target value and *l* is the total number of input samples. In SVR, the goal is to find a function *f*(**x**), i.e., an optimal hyperplane, which has at most *ε* deviation from the actually obtained target *y*_*i*_ for all the training data as flat as possible. The form of functions is denoted as

$$\begin{array}{c} f\left(x\right)=\left(\omega ,\Phi \left(x\right)\right)+b\;with\;\Phi :R^{n}\to F,\omega \in F \end{array}$$ (1)

where Φ(⋅) is a nonlinear mapping by which the input data **x** is mapped into a high dimensional space *F*, (⋅, ⋅) denotes the dot product in space *F*. Eq (1) can be transformed into the following convex constrained optimization problem by introducing the non-negative slack variables *ξ*_*i*_ and $\xi_{i}^{*}$ to cope with the otherwise infeasible constraints

$$\begin{array}{c} \begin{array}{c} min\Gamma \left(\omega ,\xi ,\xi^{*}\right)=\frac{1}{2}∥\omega {∥}^{2}+C\sum_{i=1}^{l}\left(\xi_{i}+\xi_{i}^{*}\right)\\ s.t.\left(\omega ,\Phi \left(x_{i}\right)\right)+b-y_{i}⩽\varepsilon +\xi_{i}\\ y_{i}-\left(\omega ,\Phi \left(x_{i}\right)\right)-b⩽\varepsilon +\xi_{i}\\ \xi_{i},\xi_{i}^{*}⩾0,\;i=1,2,\ldots ,l \end{array} \end{array}$$ (2)

thereinto, *C* > 0, with *C* being the penalty parameter. *ξ*_*i*_, $\xi_{i}^{*}$ are slack variables introduced in order to allow a certain error [28–32]. *ξ* is also a parameter of the *ε*-insensitive loss function, where *ε* is called the tube size [33]. The greater the value of *C* is, the greater the penalty for data points beyond the *ε* deviation, which determines the balance between the degree of smoothness of the function and the number of sample points beyond *ε* deviation. To find the upper bound of a convex quadratic programming problem, Lagrangian function is applied:

$$\begin{array}{c} \begin{array}{c} l\left(\omega ,\xi_{i},\xi_{i}^{*}\right)=\frac{1}{2}∥\omega {∥}^{2}+C\sum_{i=1}^{l}\left(\xi_{i}+\xi_{i}^{*}\right)\\ -\sum_{i=1}^{l}\alpha_{i}\left(\varepsilon +\xi_{i}-y_{i}+\left(\omega \cdot x_{i}\right)+b\right)\\ -\sum_{i=1}^{l}\alpha_{i}^{*}\left(\varepsilon +\xi_{i}+y_{i}-\left(\omega \cdot x_{i}\right)-b\right)-\sum_{i=1}^{l}\left(\eta_{i}\xi_{i}+\eta_{i}^{*}\xi_{i}^{*}\right) \end{array} \end{array}$$ (3)

thereinto, *α*_*i*_, $\alpha_{i}^{*}$, *η*_*i*_, $\eta_{i}^{*}$ are the Lagrange multiplier. The optimization problem can be obtained as follows:

$$\begin{array}{c} \begin{array}{c} min_{\alpha ,\alpha^{*}}\;\frac{1}{2}\sum_{i=1}^{l}\sum_{j=1}^{l}\left(\alpha_{i}-\alpha_{i}^{*}\right)\left(\alpha_{j}-\alpha_{j}^{*}\right)\left\langle \Phi \left(x_{i}\right),\Phi \left(x_{j}\right)\right\rangle +\varepsilon \sum_{i=1}^{l}\left(\alpha_{i}+\alpha_{i}^{*}\right)\\ -\sum_{i=1}^{l}y_{i}\left(\alpha_{i}-\alpha_{i}^{*}\right)\\ s.t.\sum_{i=1}^{l}\left(\alpha_{i}-\alpha_{i}^{*}\right)=0\\ 0⩽\alpha_{i}⩽C\\ 0⩽\alpha_{i}^{*}⩽C \end{array} \end{array}$$ (4)

where $\alpha_{i}^{*}$ is the nonnegative Lagrange multiplier that can be obtained by solving the convex quadratic programming problem. By exploiting the Karush-Kuhn-Tucker (KKT) conditions of the primal optimization problem [34–36], we can get the equation $\alpha_{i}^{*}\alpha_{j}^{*}=0$, which means that both of the multipliers $\alpha_{i}^{*}$ and $\alpha_{j}^{*}$ equal to zero, or one of multipliers is zero and $\left(\alpha_{i}^{*}-\alpha_{i}^{*}\right)$ is nonzero. The data samples with non-vanishing Lagrange multipliers are called the support vectors inside or outside the *ε*-insensitive tube [33].

The regression estimation function can be obtained by learning as follows:

$$\begin{array}{c} \begin{array}{c} f\left(x\right)=\sum_{x_{i}\in SV}\left(\alpha_{i}-\alpha_{i}^{*}\right)K\left(x_{i},x\right)+b \end{array} \end{array}$$ (5)

thereinto,

$$\begin{array}{c} \begin{array}{c} b=\frac{1}{N_{NSV}}\{\sum_{0<\alpha_{i}<C}\left[y_{i}-\sum_{x_{j}\in SV}\left(\alpha_{j}-\alpha_{j}^{*}\right)K\left(x_{j},x_{i}\right)-\varepsilon \right]+\\ \sum_{0<\alpha_{i}^{*}<C}\left[y_{i}-\sum_{x_{j}\in SV}\left(\alpha_{j}-\alpha_{j}^{*}\right)K\left(x_{j},x_{i}\right)+\varepsilon \right]\} \end{array} \end{array}$$ (6)

where *N*_*NSV*_ represents the number of standard support vectors. *K*(**x**_*i*_, **x**_*j*_) is defined as the kernel function. According to Hilbert-Schmidt principle, when kernel function matches Mercer conditions, that is, for any given function *g*(*x*), if $\int_{a}^{b}g^{2}\left(x\right)dx$ is limited, the value of the kernel is equal to the dot product of two vectors **x**_*i*_ and **x**_*j*_ in the feature space Φ(**x**_*i*_) and Φ(**x**_*j*_), i.e., *K*(**x**_*i*_, **x**_*j*_) = 〈Φ(**x**_*i*_), Φ(**x**_*j*_)〉 [33].

We choose here the Gauss radial basis function as kernel function.

$$\begin{array}{c} \begin{array}{c} K\left(x_{i},x_{j}\right)=\exp\left(-\frac{∥x-x_{i}{∥}^{2}}{\sigma^{2}}\right), \end{array} \end{array}$$ (7)

where *σ* is the kernel parameter.

### Adaptive genetic algorithm

Genetic Algorithm (GA) derives from the computer simulation study of biological system [37], which has been widely used function optimization, combinatorial optimization, job shop scheduling problems [38], complex network clustering, pattern mining [39–41]. However, there are still some disadvantages, the most obvious disadvantages are the low efficiency and easy to fall into local optimum [42, 43].

In 2000, adaptive Genetic Algorithm (AGA) [44] is proposed, which improves the performance of traditional GA to some extent. After that, adaptive GA is improved by involving certain intelligent strategies, including crossover to avoid inbreeding, crossover probability associated with the number of evolution and regulating adaptive mutation probability [45]. The formula which is only related to the number of evolution for cross-probabilistic computing is as follows:

$$\begin{array}{c} m_{tmp}=P_{c,max}*2^{-\frac{t}{T_{Gen}}} \end{array}$$ (8)

$$\begin{array}{c} P_{c}\left(t\right)=\{\begin{array}{ccc} m_{tmp} & , & m_{tmp}>P_{c,min}\\ P_{c,min} & , & m_{tmp}⩽P_{c,min} \end{array} \end{array}$$ (9)

In the formula, *m*_*tmp*_ is an intermediate variable for calculation, *T*_*Gen*_ is the maximum evolutionary number preset, *t* is the current evolutionary number (0 ≤ *t* ≤ *T*_*Gen*_), *P*_*c*, *max*_ is the largest crossover probability preset, *P*_*c*, *min*_ is the smallest crossover probability preset, and *P*_*c*_(*t*) is the crossover probability of current population.

The formula of adaptive mutation probability related to the number of genetic evolution and individual fitness is as follows:

$$\begin{array}{c} m_{tmp}=\exp[-|\frac{f_{max}-f\left(x_{i}\right)}{f_{max}}\left|\right]\cdot \frac{1}{1+\frac{t}{T_{Gen}}}\cdot P_{m,max} \end{array}$$ (10)

$$\begin{array}{c} P_{m}\left(t\right)=\{\begin{array}{ccc} m_{tmp} & , & m_{tmp}>P_{m,min}\\ P_{m,min} & , & m_{tmp}⩽P_{m,min} \end{array} \end{array}$$ (11)

In the formula, *P*_*m*, *max*_ is the largest mutation probability preset, *P*_*m*, *min*_ is the smallest mutation probability preset, *f*(**x**_*i*_) is the fitness value of individual **x**_*i*_, *f*_*max*_ is the maximum value of fitness in current populations, *P*_*m*_(*t*) is the mutation probability of individual **x**_*i*_ in current population [45].

## The mathematic model and data experiments

**Fig 1:**
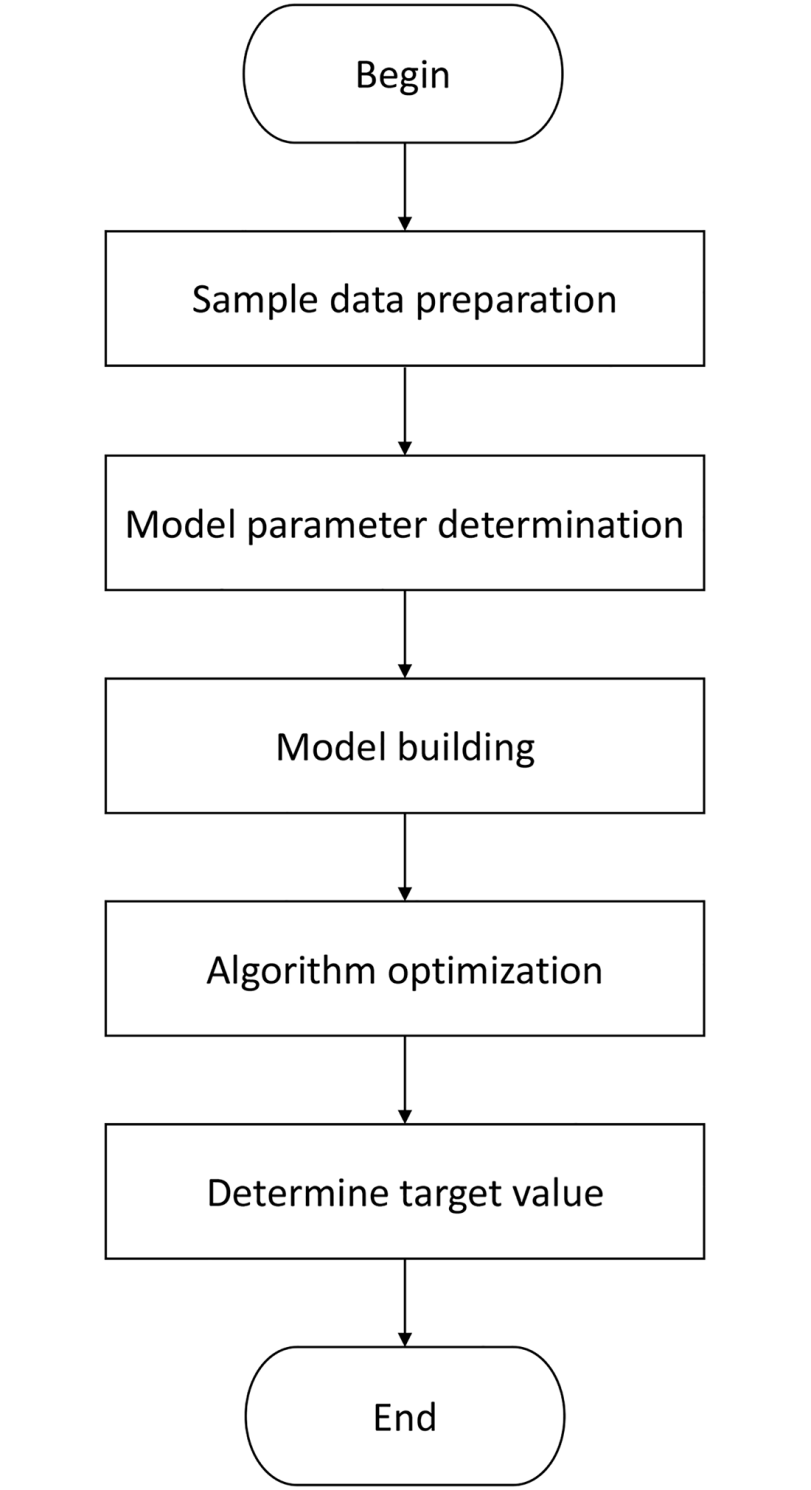
Main work flow chart.

In this section, it starts by selecting probable elements from original data, and then the values of two important parameters of the model are determined. After that, the mathematic model based on SVR is built to describe the relationship between Welan gum products and experimental conditions. With the model, AGA is applied to find the optimal sample point of the model, which corresponds to a class of potential optimal experimental conditions to maximize the production of Welan gum. The flowchart is shown in Fig 1.

### The mathematic model

#### Data preparation

Before building the mathematic model for describing the relationship between Welan gum production and experimental conditions, it needs to normalize the data. SVR mainly deals with the nonlinear problems, so the magnitude of the eigenvalues of the samples should be different greatly, the results will be greatly affected without normalizing samples. Besides, normalizing samples can avoid the small weight of the model and leading to the instability of the numerical calculation, so that the parameter optimization can converge at a faster speed and the accuracy of the model can be improved. The normalized formula used in our method is as follows:

$$\begin{array}{c} y=\frac{\left(y_{max}-y_{min}\right)\cdot \left(x-x_{min}\right)}{x_{max}-x_{min}}+y_{min}, \end{array}$$ (12)

where *x* is the original data, *y* is the normalized data, *x*_*min*_ is the minimum of the original data, *x*_*max*_ is the maximum of the original data, *y*_*min*_ is the minimum of the normalized data, *y*_*max*_ is the maximum of the normalized data. The value of *y*_*min*_ is set to be 0 and the value of *y*_*max*_ to be 1. The normalized data is shown in Tables 1 and 2 below:

**Table 1:** Sample data before normalization.

	glucose (g/L)	yeast (g/L)	KH_2_PO_4_ (g/L)	MgSO_4_ (g/L)	liquid volume (ml)	PH value	temperature (°C)	rotational speed(rpm)	inoculation amount	production (g/L)
1	40	2	5	0.1	50	10	28	150	5	0.9084
2	40	2	5	0.1	50	2	28	150	5	1.1484
3	40	2	5	0.1	50	3	28	150	5	1.6588
4	40	2	5	0.1	50	9	28	150	5	1.914
5	40	2	5	0.1	50	4	28	150	5	2.9348
6	60	10	5	0.1	50	7	32.5	175	5	3.08
7	40	2	5	0.1	50	5	28	150	5	4.0832
8	40	2	5	0.1	50	5.5	28	150	5	4.5936
9	40	2	5	0.1	50	8	28	150	5	6.2496
10	60	9	5	0.1	50	7	32.5	175	5	6.29
11	10	2	5	0.1	50	7	32.5	175	5	6.75
12	40	2	5	0.1	50	6	28	150	5	8.1664
13	60	8	5	0.1	50	7	32.5	175	5	8.7
14	20	2	5	0.1	50	7	32.5	175	5	9.23
15	40	2	5	0.1	50	6.8	28	150	1	10.73
16	40	2	5	0.1	50	7.5	28	150	5	10.9084
17	40	2	5	0.1	50	6.8	28	150	10	11.52
18	40	2	5	0.1	50	6.8	28	150	8	12.05
19	40	2	5	0.1	50	6.8	28	150	7	12.28
20	40	2	5	0.1	50	6.8	28	150	3	12.68
21	60	1	5	0.1	50	7	32.5	175	5	12.8
22	40	2	5	0.1	50	7	32.5	125	5	12.982
23	40	2	5	0.1	50	6.8	28	150	6	13.45
24	40	2	5	0.1	50	6.5	28	150	5	14.036
25	60	7	5	0.1	50	7	32.5	175	5	14.31

**Table 2:** Sample data after normalization.

	glucose (g/L)	yeast (g/L)	KH_2_PO_4_ (g/L)	MgSO_4_ (g/L)	liquid volume (ml)	PH value	temperature (°C)	rotational speed(rpm)	inoculation amount	production (g/L)
1	0.375	0.1111	1	0	0.25	1	0.3	0.25	0.4444	0
2	0.375	0.1111	1	0	0.25	0	0.3	0.25	0.4444	0.005777
3	0.375	0.1111	1	0	0.25	0.125	0.3	0.25	0.4444	0.018064
4	0.375	0.1111	1	0	0.25	0.875	0.3	0.25	0.4444	0.024207
5	0.375	0.1111	1	0	0.25	0.25	0.3	0.25	0.4444	0.04878
6	0.625	1	1	0	0.25	0.625	0.75	0.5	0.4444	0.052275
7	0.375	0.1111	1	0	0.25	0.375	0.3	0.25	0.4444	0.076425
8	0.375	0.1111	1	0	0.25	0.4375	0.3	0.25	0.4444	0.088711
9	0.375	0.1111	1	0	0.25	0.75	0.3	0.25	0.4444	0.128575
10	0.625	0.8889	1	0	0.25	0.625	0.75	0.5	0.4444	0.129547
11	0	0.1111	1	0	0.25	0.625	0.75	0.5	0.4444	0.14062
12	0.375	0.1111	1	0	0.25	0.5	0.3	0.25	0.4444	0.174716
13	0.625	0.7778	1	0	0.25	0.625	0.75	0.5	0.4444	0.187561
14	0.125	0.1111	1	0	0.25	0.625	0.75	0.5	0.4444	0.20032
15	0.375	0.1111	1	0	0.25	0.6	0.3	0.25	0	0.236428
16	0.375	0.1111	1	0	0.25	0.6875	0.3	0.25	0.4444	0.240723
17	0.375	0.1111	1	0	0.25	0.6	0.3	0.25	1	0.255445
18	0.375	0.1111	1	0	0.25	0.6	0.3	0.25	0.7778	0.268203
19	0.375	0.1111	1	0	0.25	0.6	0.3	0.25	0.6667	0.27374
20	0.375	0.1111	1	0	0.25	0.6	0.3	0.25	0.2222	0.283369
21	0.625	0	1	0	0.25	0.625	0.75	0.5	0.4444	0.286258
22	0.375	0.1111	1	0	0.25	0.625	0.75	0	0.4444	0.290639
23	0.375	0.1111	1	0	0.25	0.6	0.3	0.25	0.5556	0.301905
24	0.375	0.1111	1	0	0.25	0.5625	0.3	0.25	0.4444	0.316011
25	0.625	0.6667	1	0	0.25	0.625	0.75	0.5	0.4444	0.322607

Without losing the generality, all 67 samples collected from Welan gum producing experiments are classified according to the production, which are divided into three types: high, middle and low level production. Specifically, productions between 0g/L and 5g/L belong to low level production data, in total 8 groups; productions between 5g/L and 20g/L are in medium level, in total 39 groups; productions more than 20g/L are in high level, in total 20 groups.

Each time the model data is taken, the order of the samples within each yield is randomly arranged, For each level data groups, the first 70% of each type data is used as training data, the 30% data left are used as the testing data.

**Fig 2:**
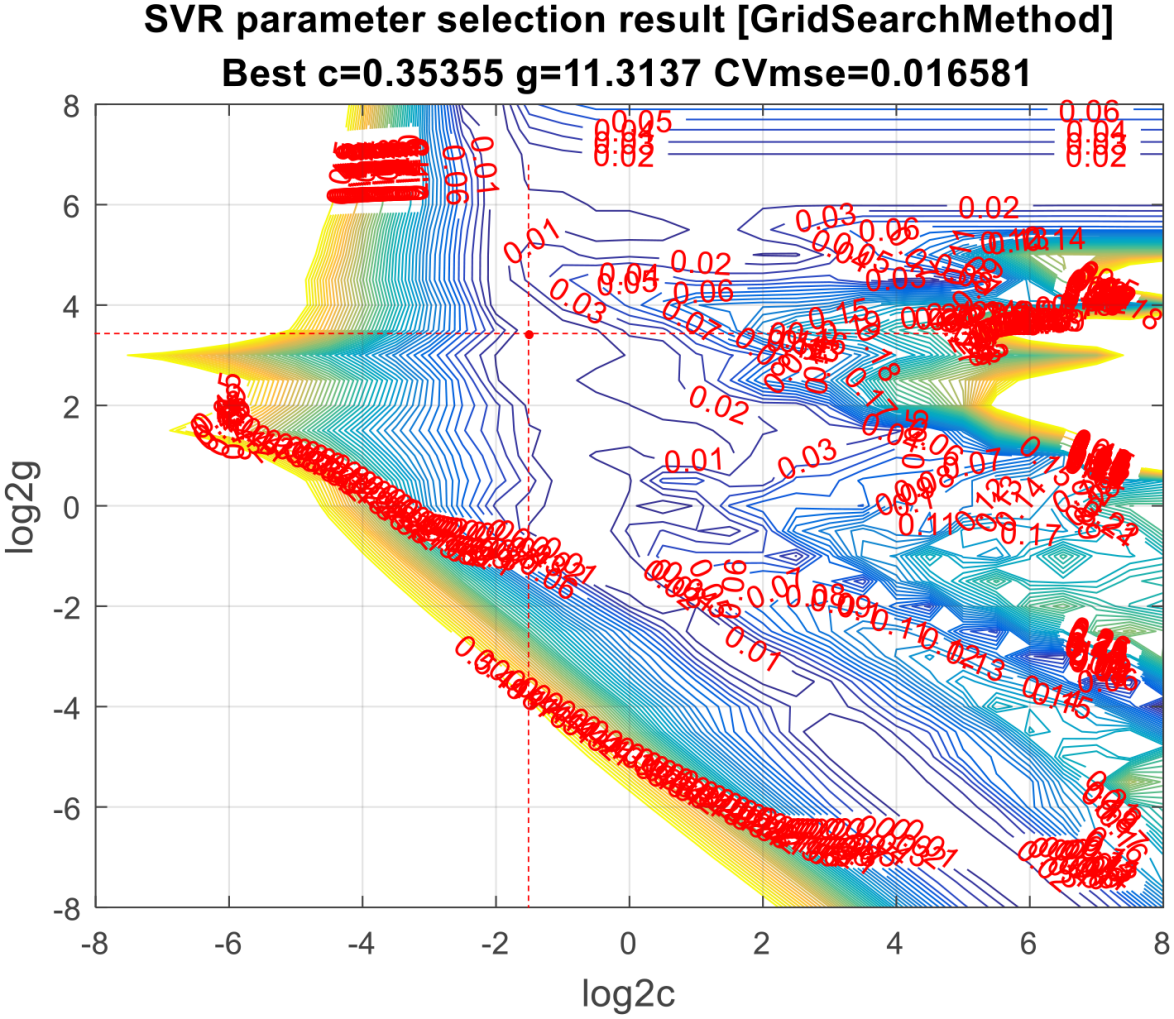
SVR parameter selection result[GridSearchMethod].

Before building the mathematic model, it is necessary to determine the values of two parameters, namely penalty factor parameters (c) and kernel function parameters (g). Here, grid search method is used to determine the optimal values of the two parameters. The result is shown in Fig 2 below:

In the above figure of contour line, two red dotted lines are represented separately the optimal values of the two parameters. The intersection of two lines, that is, the red point in the figure represents the value of the “CVmse”. The CVmse means that the mean of the squares of the difference between the predicted value and the true value under the 5-fold cross validation.

**Fig 3:**
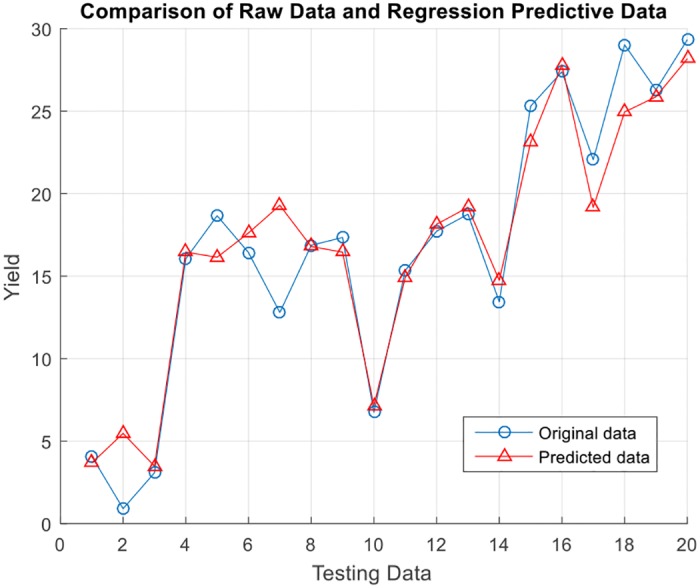
Comparison of raw data and regression predictive data.

**Fig 4:**
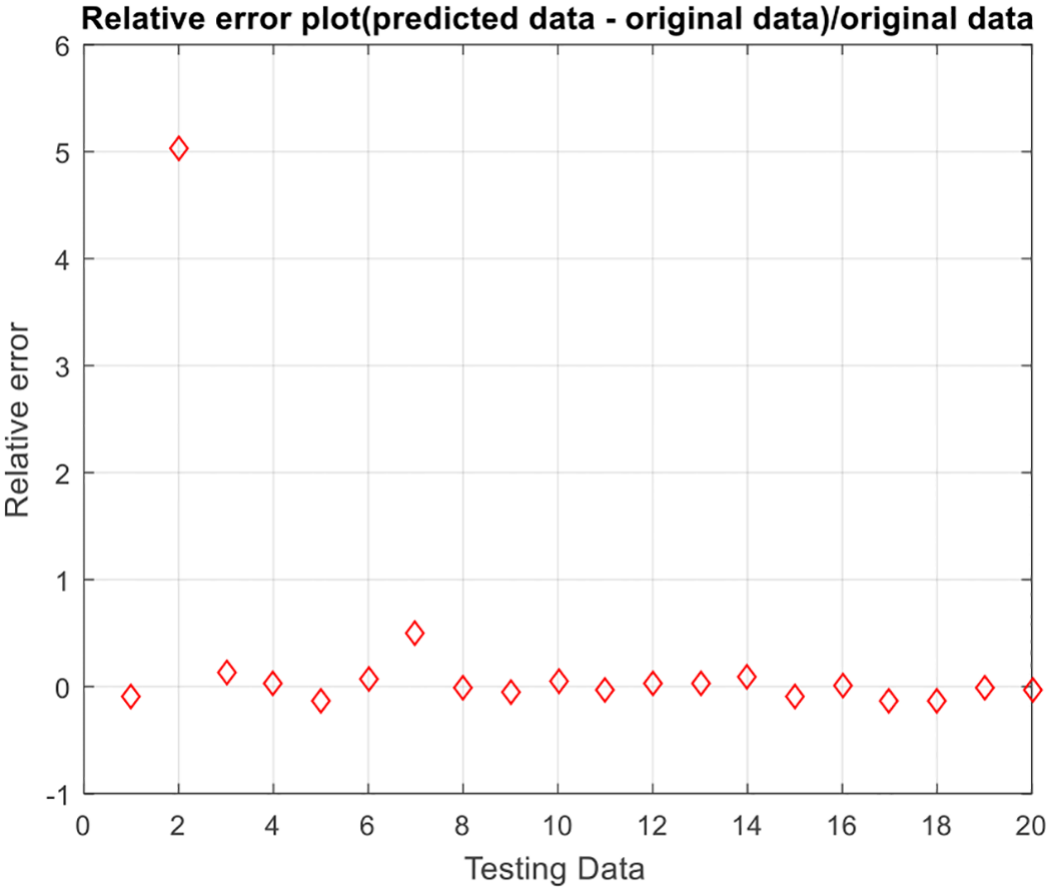
Relative error plot.

After the values of the parameters are determined, the training data and testing data are determined according to the selection of the aforementioned method. The index of the accuracy of the model is reflected in the square of correlation coefficient. The diagrams in Figs 3 and 4 reflect the model’s prediction of the testing data and the relative error.

### Finding optimal experimental conditions by AGA

With the mathematical model constructed, an improved AGA is used to find experimental conditions for optimal production. The process has the following steps.

**Step 1**: Initialize the population and encode the individuals.

Each sample is related to nine variables, so we consider the nine variables as nine genes that make up a chromosome. For example, encode [glucose, yeast, KH_2_PO_4_, MgSO_4_, fluid volume, PH value, temperature, rotational speed, inoculation amount] to [*x*_1_, *x*_2_, *x*_3_, *x*_4_, *x*_5_, *x*_6_, *x*_7_, *x*_8_, *x*_9_], where *x*_1_ ∈ [5, 95], *x*_2_ ∈ [1, 10], *x*_3_ ∈ [1, 6], *x*_4_ ∈ [0.1, 1], *x*_5_ ∈ [25, 125], *x*_6_ ∈ [2, 12], *x*_7_ ∈ [25, 35], *x*_8_ ∈ [125, 250], *x*_9_ ∈ [1, 10].

**Step 2**: Select good individuals based on the fitness values.

**Step 3**: Perform crossover operation. From the first individual in the population, the corresponding crossover probability of the individual is calculated, denoted as cross_rate. We randomly generate a random number between 0 and 1, denoted as rand_num. If the value of rand_num is less than cross_rate, the individual is performed crossover operation. That is, two integers between 1 and 9 are randomly generated, where the smaller number is the starting position of the crossed chromosome, the larger number is the ending position, the chromosome of the individual is exchanged with the chromosome of the next adjacent individual, in the range from the starting position to the termination position. In addition, if the i-th individual did not perform the crossover operation, the above-described process is repeated for the i+1-th individual; if the i-th individual performed the crossover operation, the above-described process is repeated for the i+2-th.

**Step 4**: Perform mutation operation. From the first individual in the population, the corresponding mutation probability of the individual is calculated, denoted as mutate_rate. We randomly generate a random number between 0 and 1, denoted as rand_num. If the value of rand_num is less than mutate_rate, the individual is performed mutation operation. That is, an integer between 1 and 9 is randomly generated as the location of the gene that needs to be mutated, regenerate the gene at the location.

**Step 5**: The new individuals generated by the above operations constitute the new population, and go to step 2.

Repeat these steps until we find the optimal individual.

**Fig 5:**
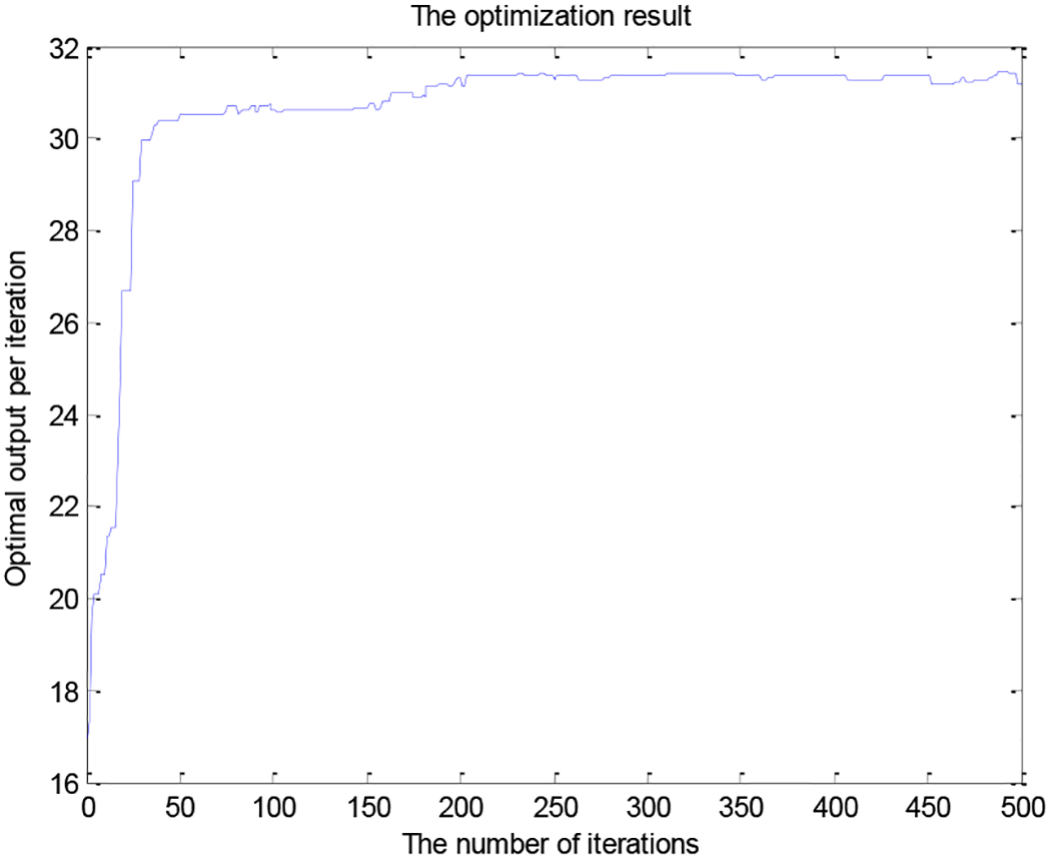
The optimization result.

The size of initial population is set to be 300, that is there are 300 individuals, the number of iterations is 500. The selection operator is roulette selection method, which is also known as the proportional selection operator. The basic idea is that the probability of each individual selected is proportional to its fitness value.

$$\begin{array}{ccc} & & P\left(x_{i}\right)=\frac{f(x_{i})}{\sum_{i=1}^{K}f\left(x_{i}\right)}, \end{array}$$ (13)

where *P*(*x*_*i*_) is the selection probability of individual *x*_*i*_, K is the population size. The value of parameter *P*_*c*,*min*_ is set to be 0.6, *P*_*c*,*max*_ to be 0.9, *P*_*m*,*max*_ to be 0.1 and *P*_*m*,*max*_ to be 0.001. The search results are shown in Fig 5.

**Fig 6:**
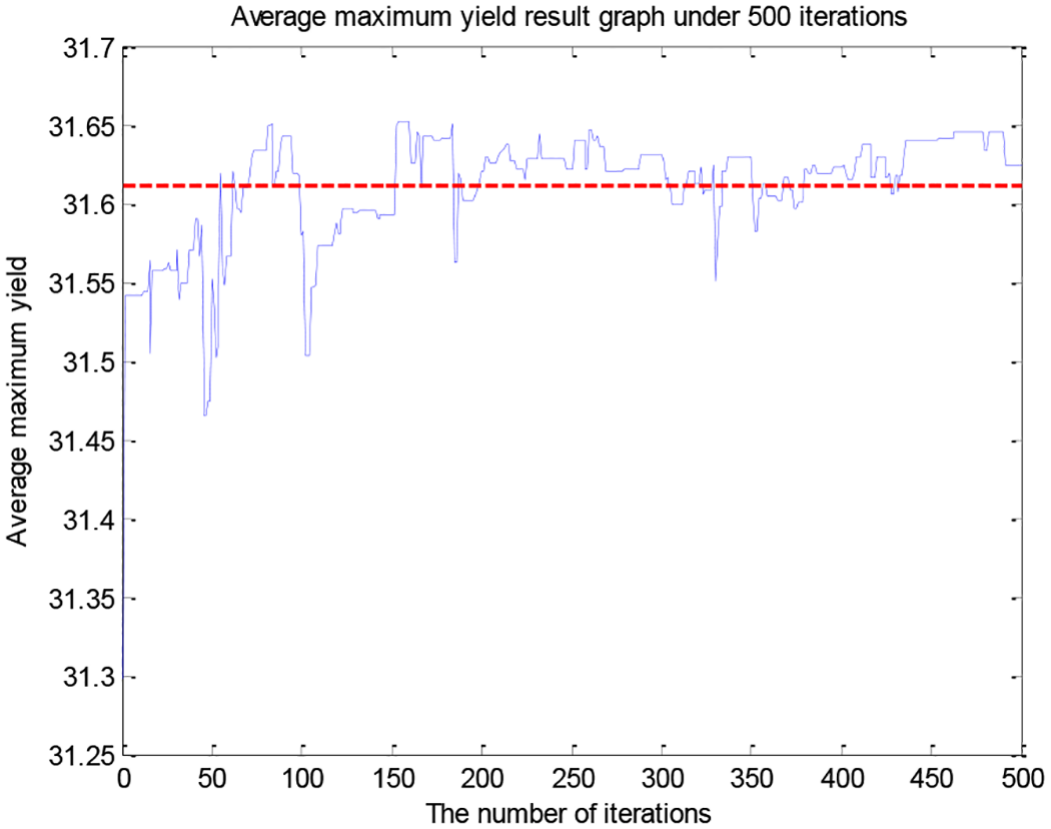
The average maximum yield result graph under 500 iterations.

To improve the accuracy and further reduce the range of the nine gene variables. We made the following changes by observing the genetic variables of samples with productions higher than 30g/L, which is *x*_1_ ∈ [55, 60], *x*_2_ ∈ [2.5, 3.1], *x*_3_ ∈ [5, 5.5], *x*_4_ ∈ [0.1, 0.3], *x*_5_ ∈ [48, 51.5], *x*_6_ ∈ [6.7, 7.15], *x*_7_ ∈ [32, 33], *x*_8_ ∈ [176, 179], *x*_9_ ∈ [4.85, 5.15]. The average maximum fitness value of data experiments with 500 iterations each time is shown in Fig 6.

## Results

The accuracy of the established mathematic model is 88.36%, the optimal medium composition ratio is shown in Table 3 below:

**Table 3:** The optimal medium composition ratio.

glucose (g/L)	yeast (g/L)	KH_2_PO_4_ (g/L)	MgSO_4_ (g/L)	liquid volume (ml)	PH value	temperature (°C)	rotational speed(rpm)	inoculation amount
55.26	2.89	5.23	0.1	49.8	7.01	32.53	177.51	5

The maximum production of Welan gum is 31.65g/L.

This hybrid computational method, which combines with SVM and AGA, has the intelligent learning ability and can overcome the limitation of large-scale biotic experiments [46–51]. A mathematic model of predicting production of Welan gum from experimental conditions with accuracy rate 88.36% is obtained, a class of optimized experimental conditions is designed to produce Welan gum 31.65g/L. Comparing the best results in chemical experiment 30.63g/L, the predicted production can be improved by 3.3%.

## Conclusion

We focused on building a mathematic model of Welan gum, the nine factors which contribute the experimental conditions of producing Welan gum as preparative optimization indicators. The nine factors include glucose, yeast, KH_2_PO_4_, MgSO_4_, fluid volume, PH value, temperature, rotational speed and inoculation amount. A hybrid computational method combined with SVM and AGA is proposed. Through the training of sample data, a mathematic model of predicting production of Welan gum from experimental conditions is obtained. We find the optimal sample point in the sample space, i.e. a class of optimized experimental conditions. This hybrid computational method has a good learning ability, which can avoid the high cost problem caused by large-scale biological experiments. It also overcomes the “mature” defects of traditional Genetic Algorithm. The result provides a potential experimental conditions by data mining to improve the production of Welan gum in the lab.

For further research, neural-like computing models, e.g., spiking neural P systems [52] can be used for optimization of Welan gum production. As well, some recently developed data processing and mining methods, such as the speculative approach to spatial-temporal efficiency for multi-objective optimization in cloud data and computing [53], privacy-preserving smart similarity search methods in simhash over encrypted data in cloud computing [53], k-degree anonymity with vertex and edge modification algorithm [54], kernel quaternion principal component analysis for object recognition [55], might be used for optimizing experimental conditions of Welan gum. In the aspect of data preparation, decision tree [56] can be used to deal with the missing attribute value of some samples in dataset.
